# Synthesis and biological evaluation of diclofenac acid derivatives as potential lipoxygenase and α-glucosidase inhibitors

**DOI:** 10.1098/rsos.240543

**Published:** 2024-11-20

**Authors:** Asma Sardar, Obaid-ur-Rahman Abid, Wajid Rehman, Liaqat Rasheed, Mohammed M. Alanazi, Saima Daud, Muhammad Rafiq, Abdul Wadood, Muhammed Shakeel

**Affiliations:** ^1^ Department of Chemistry, Hazara University, Mansehra 21300, Pakistan; ^2^ Department of Pharmaceutical Chemistry, College of Pharmacy, King Saud University, Riyadh 11451, Saudi Arabia; ^3^ South China University of Technology School of Chemistry and Chemical Engineering, Guangzhou, People’s Republic of China; ^4^ Department of Biochemistry, Abdul Wali Khan University, Mardan, Khyber Pakhtunkhwa, Pakistan; ^5^ State Key Laboratory of Chemical Resource Engineering, Beijing University of Chemical Technology, Beijing 100029, People’s Republic of China

**Keywords:** azole, α-glucosidase, thiosemicarbazide, cytotoxicity, 15-lipoxygenase and molecular docking

## Abstract

Inflammation is a complex physiological response associated with the onset and progression of various disorders, including diabetes. In this study, we synthesized a series of diclofenac acid derivatives and evaluated their potential anti-diabetic and anti-inflammatory activities. The compounds were specifically assessed for their ability to inhibit 15-lipoxygenase (15-LOX) and α-glucosidase enzymes. The structures of synthesized derivatives were confirmed through ^1^H nuclear magnetic resonance (NMR), ^13^C-NMR and high-resolution mass spectrometry (electron ionization) analysis. All these synthesized derivatives exhibited varying degrees of inhibitory activity against LOX, when compared with standard drugs, compounds **5a** (half-maximal inhibitory concentration (IC_50_) 14 ± 1 µM), **5b** (IC_50_ 61 ± 1 µM) and **7c** (IC_50_ 67 ± 1 µM) showed good activity against the LOX enzyme. While the α-glucosidase inhibitory results revealed that most of the compounds exhibited significant activity when compared with the standard drug acarbose (376 ± 1 µM). The most potent compounds as α**-**glucosidase inhibitors were **7b** (3 ± 1 µM), **4b** (5 ± 1 µM), **7a** (7 ± 1 µM) and **8b** (11 ± 1 µM). All these active compounds were found to be least toxic and maintained the mononuclear cells viability at 96–97% compared with that of controls as determined by multi-transaction translator assay. Molecular docking studies further reiterated the significance of these ‘lead’ compounds with great potential against the target enzymes in the process of drug discovery.

## 1. Introduction

Diabetes mellitus, a chronic metabolic disorder characterized by elevated blood glucose levels, represents a significant global health challenge with profound implications for public health and patient well-being [1–6]. Central to its pathophysiology is a complex interplay of genetic, environmental and lifestyle factors that converge to disrupt glucose homeostasis and insulin signalling, leading to hyperglycaemia and a myriad of associated complications. Among these, chronic inflammation emerges as a hallmark feature, contributing not only to the progression of diabetes but also to the development of its vascular and microvascular complications [7–10].

In recent years, the role of inflammation in diabetes has garnered increasing attention, with growing evidence implicating various inflammatory mediators and pathways in the pathogenesis of the disease [3,4]. One such mediator is the enzyme 15-lipoxygenase (15-LOX), a key player in the biosynthesis of lipid mediators involved in inflammation and immune regulation [11]. Emerging research suggests that dysregulation of 15-LOX activity may contribute to the chronic low-grade inflammation observed in diabetes, further exacerbating insulin resistance, β-cell dysfunction and the progression of diabetic complications [12–17].

Effective treatment approaches for diabetes mellitus include the use of α-glucosidase inhibitors, which help in delaying and reducing postprandial blood glucose levels. α-Glucosidase is a carbohydrate-metabolizing enzyme that has a role in diabetes, cancer and viral infections [8,18–20]. α-Glucosidase is a therapeutic agent used for biological inhibitors. Anti-diabetic medicines, including acarbose, voglibose and miglitol compete for α-glucosidase in the small intestine, interrupting carbohydrate breakdown and improving postprandial hyperglycaemia. Continuous use of these medications, on the other hand, might result in a variety of side effects, including abdominal pain and diarrhoea [21,22]. As a result, new α-glucosidase inhibitors without these issues must be developed. Recent research revealed that 15-LOX inhibitors play an important role in medicinal chemistry and drug innovation [23–25]. No drug is marketed as 15-LOX inhibitor in the market except Zileuton which is used in the treatment of asthma but is accompanied by side effects including nausea, sinusitis and pharyngolaryngeal pain. Therefore, there is always a need to find drugs with less side effects and improved efficacy. A literature survey has shown that the inhibition of LOX by heterocyclic compounds can be regarded as a way forward in the discovery of ‘lead’ compounds as anti-inflammatory agents [26–29].

**Figure 1.**
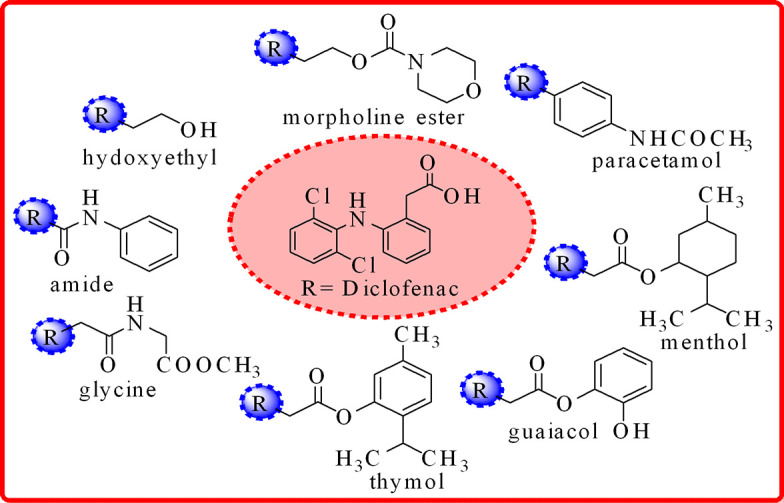
Structures of diclofenac acid prodrugs.

Diclofenac acid (2-(2-((2,6-dichlorophenyl)amino) phenyl) acetic acid) is a non-steroidal anti-inflammatory drug (NSAID), the most prescribed drug all over the world, which ranked 30th in the top 200 drugs [12]. NSAIDs, including diclofenac, are inherently responsible for gastrointestinal, renal and hepatic adverse effects. Long-term NSAID therapy is commonly associated with several adverse effects, notably upper gastrointestinal irritation, ulceration and, in severe cases, hepatic coma and cardiovascular disorders [30–33]. These risks can pose significant health concerns and, in extreme situations, even lead to fatal outcomes [34,35]. To reduce and limit these gastrointestinal complications, the free carboxylate moiety of these NSAIDs has been transformed into certain prodrugs [36]. For example, diclofenac acid has been transformed as prodrugs into ester [37], amides [38,39] and also incorporated with amino acids like glycine [40], with various drugs like paracetamol [41] and with anti-oxidant agents like menthol, thymol and guaiacol [42] (figure 1).

Literature studies described some NSAIDs derivatives as biologically potent molecules [43–46]. Keeping in view the above specifics, there is still a need for more potent inhibitors with fewer side effects and more efficacies against the 15-LOX and α-glucosidase enzymes. Therefore, we have synthesized diclofenac acid derivatives containing thiosemicarbazides and azoles in the present studies and evaluated them for their soybean 15-LOX and α-glucosidase inhibition studies. The novelty of our work lies in its significant advancement in diabetes drug discovery. Unlike previous studies, our research offers novel compounds with targeted inhibitory activity against 15-LOX or α-glucosidase enzymes. Although none of the compounds demonstrated dual inhibition, several compounds showed significant activity against one of the two targets. This approach addresses unmet therapeutic needs by focusing on key pathways implicated in diabetes pathogenesis. The findings highlight the potential of these compounds in developing more effective therapies for managing diabetes and related conditions, offering versatility in addressing various aspects of diabetes pathology. Overall, our research represents a pioneering effort in the field, paving the way for the development of more effective and multifaceted treatments for diabetes.

## 2. Experimental

### 2.1. Material and methods

All of the chemicals and solvents were obtained from Sigma-Aldrich and used directly without any purification. Mononuclear cells were isolated from the blood samples taken from healthy volunteers. Diclofenac acid was purchased from Alfa Aesar. Bruker Avance 400 and 500 MHz spectrometers were used to simulate the ^1^H and ^13^C nuclear magnetic resonance (NMR) spectra, were captured with tetramethylsilane (TMS) serving as an internal reference. On a Finnegan MAT-311A mass spectrometer, high-resolution mass spectrometry using electron ionization (HRMS (EI)) data were recorded. Using the Stuart melting point apparatus, melting points in open capillaries were determined (SMP10). All reactions were monitored by using thin layer chromatography (TLC) plates that were pre-coated with silica gel 60F254.

### 2.2. General method for the synthesis of diclofenac methyl ester (**2**)

Diclofenac methyl ester was synthesized by a previously reported method [8]. Ester (**2**) was prepared by the reaction of diclofenac acid/naproxen (0.02 mol; **1**) and methanol in the presence of a few drops of concentrated H_2_SO_4_. First, diclofenac acid was dissolved in dry methanol (40–45 ml) then to that reaction mixture 1.5 ml of H_2_SO_4_ was added and was refluxed for approximately 5–6 h at 65°C. The reaction progress was monitored through TLC. After completion, excess methanol evaporated, and the product was left behind with a fruity, pleasant smell. For the purification process, a separatory funnel was used. An oily layer was poured into 50 ml of water and 30 ml of dichloromethane was added. The unreacted acid was removed through extraction with a 5% sodium carbonate solvent. Then removed the organic layer, dried it using anhydrous calcium chloride. The solvent was removed through evaporation, and pure ester was left behind in solid form.

### 2.3. General method for the synthesis of diclofenac hydrazide (**3**)

For the synthesis of hydrazide (**3**), 0.01 mol of ester (**2**) was dissolved in ethanol (25 ml) and 0.02 mol of hydrazine hydrate added dropwise in the reaction mixture and refluxed with continuous stirring at 80°C for approximately 16–17 h [8]. Reaction completion was observed through TLC. Excess solvent was evaporated to get solid products, then the product was washed with water to remove the excess of hydrazine hydrate, dried and recrystallized with ethanol to obtain pure product [47,48].

### 2.4. General method for the synthesis of thiosemicarbazides (**4a–f**)

Thiosemicarbazides (**4a–f**) were synthesized through the reflux method, using an equimolar amount of hydrazides (0.01 mol; **3a,b**) and isothiocyanates (0.01 mol). The hydrazide was dissolved in 15–20 ml of ethanol, and isothiocyanates were separately dissolved in 10–15 ml ethanol, and then both the solutions were mixed and refluxed for 6–7 h at 80°C. The progress of the reaction was monitored through TLC. After reaction completion, mixture was cooled at room temperature, the solvent was evaporated and the solid left behind was washed and filtered to get the pure compound.

### 2.5. General method for the synthesis of 1,2,4-triazole-3-thiol (**5a–f**)

Thiosemicarbazide (**4a–f**; 0.0011 mol) was added portion-wise to the stirred solution of NaOH (5%, 30 ml). The reaction mixture was allowed to reflux for 3–4 h. After cooling, the reaction mixture was filtered and the filtrate was acidified with 6 N HCl to pH 2–3. The precipitated solid was filtered, washed thoroughly with water and recrystallized with ethanol.

### 2.6. General method for the synthesis of S-alkylated 1,2,4-triazoles (**6a–d**)

1,2,4-Triazole-3-thiol (**5c, 5f**; 0.002 mol) and K_2_CO_3_ (0.0025 mol) were mixed in dimethylformamide (DMF) (15–20 ml) and refluxed for approximately half an hour, then ethyl/propyl bromide (0.0021 mol) was added dropwise to reaction mixture and refluxed for 2–3 h. After completion of reaction (monitored through TLC), the reaction mixture was cooled and poured into ice-cold water, precipitates were filtered out, washed, dried and recrystallized with ethanol.

### 2.7. General method for the synthesis of 1,3,4-oxadiazoles (**7a–f**)

Various thiosemicarbazides (0.001 mol**; 4a–f**) were dissolved in ethanol and the solution of mercuric acetate (0.0013 mol) in ethanol was added dropwise. The reaction mixture was refluxed for 2–3 h at 80°C with continuous stirring and monitored through TLC. After completion of reaction, the reaction mixture was filtered. Excess of solvent was evaporated, and the resulting product was recrystallized from ethanol to obtain pure product.

### 2.8. General method for the synthesis of 1,3,4-thiadiazole (**8a–f**)

1,3,4-Oxadiazole (**8a–f**) were synthesized by the cyclization of thiosemicarbazides (**4a–f**; 0.001 mol), using mercuric acetate (0.0013 mol) in 10–15 ml ethanol as a solvent. The reaction mixture was refluxed for 2–3 h with continuous stirring at 80°C. The progress of reaction was observed through TLC. The reaction mixture was filtered, cooled at room temperature after reaction completion. The excess solvent was evaporated to obtain solid mass as product [3].

### 2.9. *In vitro* 15-lipoxygenase inhibition assay

Assay protocols for bioactivity were placed in electronic supplementary material.

### 2.10. *In vitro α*-glucosidase inhibition assay

Assay protocols for bioactivity were placed in electronic supplementary material.

### 2.11. Cellular viability assay

Assay protocols for bioactivity were placed in electronic supplementary material.

### 2.12. Molecular docking study

Molecular docking of the most active compounds against LOX and α-glucosidase enzymes was carried out using Molecular Operating Environment (MOE) software. Before docking the Protein Data Bank (PDB) structure was prepared by removing water molecules and then three-dimensional protonation was carried out. The energy minimization was carried out using a gradient of 0.05. For each compound, a total of 10 poses were generated. The Triangular Matching docking method of MOE software was used for docking study. Finally, the MOE and PyMol software were used for the interaction of protein–ligand complexes.

Further docking procedures were placed in electronic supplementary material.

## 3. Results and discussion

### 3.1. Chemistry

**Scheme 1.**
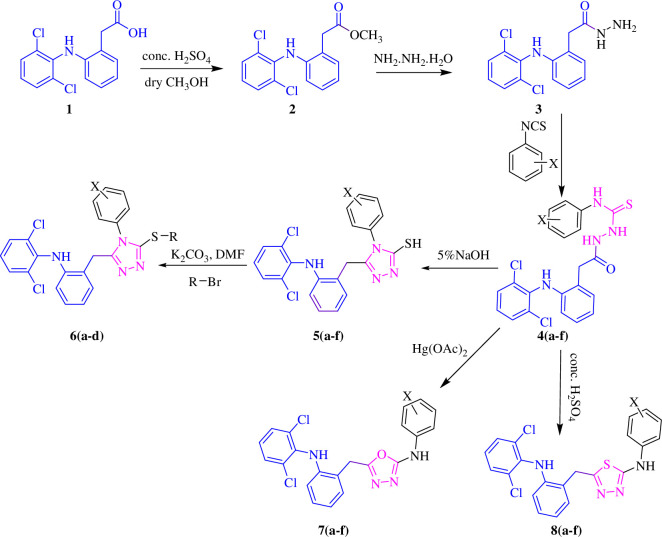
Synthesis of diclofenac acid derivatives.

The synthetic procedure is initiated by the treatment of diclofenac acid **1** with methanol in an acidic medium, which results in the formation of diclofenac ester **2**. This ester **2** was then refluxed with hydrazine hydrate, yielding the 2-(2-(2,6-dichlorophenylamino) phenyl) acetohydrazide **3.** EtOH was used as a solvent. Further, hydrazide **3** was treated with different substituted isothiocyanates to synthesize thiosemicarbazides **4 (a–f)**. ^1^H NMR analysis confirmed the formation of desired thiosemicarbazides by the appearance of signals of NH protons (NHCSNH, CONH) in the characteristic range of 11.26–9.47 ppm and Ar-H appeared in their respective range. In ^13^C NMR, the appearance of signals at 182.1–180.9 and 171.9–169.8 ppm confirmed the presence of C=O and C=S groups, respectively, and their synthesis was also confirmed by mass analysis. These thiosemicarbazides **4 (a–f)**, upon the addition of 5% NaOH, produced 5-substituted-phenyl−4H−1,2,4-triazole−3-thiol **5 (a–f)**. In ^1^H NMR spectra of 1,2,4-triazoles, the SH proton signal was found in the relevant range of 13.97–13.83 ppm, and aromatic protons were found in their respective ranges. In ^13^C NMR spectra, the appearance of signals at 168.9–168.4 and 28.7–28.4 ppm showed the C-SH and CH_2_ groups, respectively, and these characteristics peaks confirmed 1,2,4-triazole **5 (a–f)** synthesis. HRMS (EI) data also confirmed the synthesis of derived molecules. Compounds **5 (a–f)** were further treated with alkyl halides in the presence of K_2_CO_3_ in DMF to get S-alkylated 1,2,4-triazoles **6 (a–d)**. The synthesis of desired compounds was confirmed by ^1^H-NMR and ^13^C-NMR data. The alkylated protons attached to the sulphur atom appeared in their relevant range of 2.86–0.82 ppm, and aromatic protons appeared in their significant range. In ^13^C NMR spectra, the C–S and alkylated carbons are observed in the ranges of 166.6–159.2 and 34.9–12.5 ppm, respectively, confirming the synthesis of compounds **6 (a–d)**. These compounds were also confirmed by HRMS (EI) analysis. The thiosemicarbazides **4 (a–f)** upon the addition of mercuric acetate in the presence of EtOH yield substituted oxadiazole **7 (a–f)** while under stirring condition with cold sulphuric acid substituted thiadiazoles **8 (a–f)** were synthesized. These oxadiazoles and thiadiazoles were confirmed by ^1^H-NMR and ^13^C-NMR analysis. In ^1^H-NMR spectra, the signal of NH proton (attached to oxadiazole ring) was found in the appropriate range of 10.79–10.41 ppm that confirmed the oxadiazoles series **7 (a–f),** while the synthesis of thiadiazoles **8 (a–f)** was confirmed by the appearance of NH peak (attached to thiadiazoles ring) in the range of 10.25–9.72 ppm. In ^13^C-NMR spectra, C–NH peak found in the range of 160.3–159.5 ppm confirmed the oxadiazoles series **7 (a–f)** and thiadiazoles **8 (a–f)** was confirmed by the appearance of signals of C–NH (of thiadiazoles ring) in the range of 164.3–163.4 ppm. HRMS (EI) data given in the experimental section further confirmed the oxadiazoles series **7 (a–f)** and thiadiazoles **8 (a–f)** [39,41,42]. The synthetic pathway is illustrated in scheme 1 and the structures are depicted in table 1.

**Table 1.** Structures of diclofenac acid derivatives.

compd.	X	compd.	X	R
**4a**	−2F	**6a**	−4F	CH_2_CH_3_
**4b**	−3F	**6b**	−3,4 Cl	CH_2_CH_3_
**4c**	−4F	**6c**	−4F	CH_2_CH_2_CH_3_
**4d**	−2,3 Cl	**6d**	−3,4 Cl	CH_2_CH_2_CH_3_
**4e**	−2,4 Cl	**7a**	−2F	—
**4f**	−3,4 Cl	**7b**	−3F	—
**5a**	−2F	**7c**	−4F	—
**5b**	−3F	**7d**	−2,3 Cl	—
**5c**	−4F	**7e**	−2,4 Cl	—
**5d**	−2,3 Cl	**7f**	−3,4 Cl	—
**5e**	2,4 Cl	**8c**	−4F	—
**5f**	3,4 Cl	**8d**	−2,3 Cl	—
**8a**	2F	**8e**	−2,4 Cl	—
**8b**	3F	**8f**	−3,4 Cl	—

### 3.2. Biological evaluation

#### 3.2.1. *In vitro* 15-lipoxygenase inhibition studies and structure–activity relationship

The synthesized diclofenac acid derivatives were screened for their *in vitro* 15-LOX inhibitory activity and the results are given in table 2. Thiosemicarbazides were poor inhibitors (half-maximal inhibitory concentration (IC_50_) 163 ± 1 to 177 ± 1 µM), triazole bearing thiols analogues showed considerable inhibitory profiles (IC_50_ 14 ± 1 to 141 ± 1 µM), 2-arylamino-5-substituted-1,3,4-oxadiazoles showed excellent to poor inhibitory activities (IC_50_ 67 ± 1 to 172 ± 1 µM) and 2-arylamino-5-substituted-1,3,4-thiadiazoles showed remarkable to least activities (IC_50_ 97 ± 0.3 to 173 ± 1 µM) while some compounds were found with high IC_50_ value. Substitution of –F or di-Cl groups did not affect the inhibitory profiles of thiosemicarbazides as depicted in the IC_50_ inhibitory profiles. When these analogues substituted with −2F substituted **(5a)** was found to be the most potent scaffold against the targeted enzyme having an IC_50_ 14 ± 1 µM, 3 F compound **(5b)** showed excellent activity (IC_50_ 61 ± 1 µM) and **(5c)** was found with IC_50_ value greater than 250. Likewise, the substitution of 3,4-diCl **(5f)** was more active than −2,4-diCl **(5e)** which was more active than −2,3-diCl **(5d)**. It means that the mono-substituted fluorinated –N group containing analogues boost up the activity due to the positive inductive effect of fluorine, whereas less activity was observed for the −2,3-dichloro N-phenyl group compared with the related isomers, which declared that the steric hindrance due to ortho substitution lowered the inhibition [42].

**Table 2.** *α*-Glucosidase, cell viability and 15-LOX profiles of diclofenac acid derivatives.

compd.	IC_50_ (µM) ± SEM		cell viability (%) at 0.25 **mM**
	15-LOX	α-glucosidase	
**4a**	163 ± 1	53 ± 1	79 ± 2
**4b**	>250	5 ± 1	82 ± 1
**4c**	>250	53 ± 1	62 ± 2
**4d**	164 ± 1	28 ± 1	73 ± 1
**4e**	177 ± 1	87 ± 1	64 ± 2
**4f**	>250	93 ± 1	64 ± 2
**5a**	14 ± 1	202 ± 1	97 ± 2
**5b**	61 ± 1	308 ± 1	96 ± 2
**5c**	>250	170 ± 1	96 ± 2
**5d**	141 ± 1	83 ± 1	96 ± 2
**5e**	93 ± 1	92 ± 1	94 ± 1
**5f**	75 ± 1	247 ± 1	97 ± 2
**6a**	>250	116 ± 1	88 ± 2
**6b**	>250	132 ± 1	98 ± 1
**6c**	>250	92 ± 1	85 ± 1
**6d**	>250	90 ± 1	78 ± 2
**7a**	98 ± 1	7 ± 1	76 ± 1
**7b**	87 ± 1	3 ± 1	72 ± 1
**7c**	67 ± 1	85 ± 1	63 ± 1
**7d**	165 ± 1	79 ± 1	62 ± 2
**7e**	172 ± 1	61 ± 1	43 ± 1
**7f**	143 ± 1	91 ± 1	59 ± 2
**8a**	>250	99 ± 1	86 ± 2
**8b**	97 ± 1	11 ± 1	92 ± 2
**8c**	173 ± 1	55 ± 1	76 ± 1
**8d**	132 ± 1	46 ± 1	98 ± 2
**8e**	>250	NA	75 ± 1
**8f**	156 ± 1	125 ± 1	95 ± 2
quercetin	2 ± 1	—	92 ± 1
baicalein	22 ± 1	—	—
acarbose	—	376 ± 1	—
cyclophosphamide	—	—	56 ± 2
cisplatin	—	—	52 ± 2
curcumin	—	—	74 ± 2

NA = not active.

In 2-arylamino-5-substituted-1,3,4-oxadiazoles when compared with 5-substituted-phenyl-4H-1,2,4-triazole-3-thiols, −4F substituted derivative **(7c)** was more active (IC_50_ 67 ± 1 µM) than −3F substituted derivative **(7b)** (IC_50_ 87 ± 1 µM) which was more active than −2F derivative **(7a)** (IC_50_ 98 ± 1 µM). This gave us evidence that an increase in the distance of fluorine from the amino group enhanced the activity. In other words, the fight between the mesomeric effect of amino and the positive inductive effect of fluorine seems minimal by increasing the distance between the two groups. However, the substitution of –diCl groups had a small effect on the inhibitory profiles in the medium to poor range. It indicated little competition between mesomeric and inductive effects.

2-Arylamino-5-substituted-1,3,4-thiadiazoles showed remarkable to least activities, wherein, −3F substituted compound **(8b)** was more active than −4F **(8c)**. The −2F substituted compound **(8a)** was found with high IC_50_ value greater than 250. This was evident in domination of mesomeric effect of amino at 2 and 4 positions and the enhanced positive inductive effect at 3 positions. 2,3-diCl substituted compound **(8d)** was moderately more active than 3,4-diCl **(8f)**. The 2,4-diCl substituted compound was found to be inactive. It meant both amino as well as the chloro groups combined together promoted the mesomeric effect on the aryl ring [43].

#### 3.2.2. *In vitro* α-glucosidase inhibition profile and structure–activity relationship

Synthesized diclofenac acid derivatives were screened for α-glucosidase inhibitory activity (table 2). The results revealed that these derivatives showed excellent α*-*glucosidase inhibition with IC_50_ value in the range of 3.4 ± 1 to 308 ± 1 µM compared with standard drug acarbose (IC_50_ 376 ± 1 µM) except compound **8e**, which was found inactive. The most active analogue among the class was **7b** (IC_50_ value 3.4 ± 1 µM), followed by compounds **4b** (5 ± 1 µM), **7a** (7 ± 1 µM) and **8b** (11 ± 1 µM), which were better than standard.

Thiosemicarbazides **4** (**a–f**) displayed excellent inhibitory potential, with **4b** (5 ± 1 µM) and **4d** (28 ± 1 µM) being the most active compounds. Compound **4b** contains a fluoro substituent at the phenyl ring at 3-position, whereas compound **4d** has dichloro groups at the 2, 3-position. All additional compounds from the same series exhibited activity between 53 ± 1 and 92 ± 1 µM. The inhibitory activity of 1,2,4-triazoles and S-alkylated 1,2,4-triazoles was found to be moderate (83 ± 1 to 308 ± 1 µM) in comparison to thiosemicarbazides. The most effective series overall was **7** (**a–f**), where all of the compounds demonstrated excellent inhibitory potential at concentrations ranging from 3 ± 1 to 91 ± 1 µM. Compounds in this series with a fluoro substituent at the phenyl ring were more active than those with a dichloro substituent at the same phenyl ring. Compound **7b** (IC_50_ value 3 ± 1 µM) and **7a** (IC_50_ value 7 ± 1 µM) were considered excellent inhibitors due to the presence of fluoro substituents at 3 and 2 position, respectively. The 1,3,4-thiadiazoles also showed good activity appeared in the range of 11 ± 1 to 125 ± 1 µM while one compound, **8e**, was found non-active. Most potent analogue among the class is analogue **8b** (11 ± 1 µM), containing –F group in their structure. These observations suggests that all the synthesized compounds having mono-substituent as a fluoro group at 2 and 3 position of the phenyl ring showed potent inhibitory activity as compared with those compounds having dichloro substituent at the same phenyl ring, except in the case of thiosemicarbazide, in which dichloro group at 2,3-position enhanced the activity, and compounds with 1,3,4-oxadiazole moiety with fluoro substituents enhance the inhibitory activity as compared with triazole and thiadiazoles containing compounds [44,45].

#### 3.2.3. Cellular viability

Using mononuclear cells (MNCs) at a concentration of 0.25 mM as shown in the table 2, the per cent viability was calculated. The cellular viability of compounds **4 (a–f)** ranged from 62% to 82%, compounds **5 (a–f)** from 94% to 97% and compounds **6 (a–d)** from 78% to 98% against MNCs. Compounds **8 (a–f)** also maintained cellular viability between 75% ± 1% and 98% ± 2%. The most toxic compound among compounds **7(a–f)** was **7e** (43% ± 1% viability), while all the other compounds maintained cell viability between 59% ± 2% and 76% ± 1%. These findings proved that the least toxic compounds were most effective against the LOX and α*-*glucosidase enzymes. According to the results recorded, azole derivatives of diclofenac acid inhibited the LOX and α-glucosidase enzymes in *in vitro* enzyme inhibition studies differently, and the active compounds were generally the least toxic to MNCs at the specified concentration and experimental conditions.

### 3.3. Molecular docking studies

#### 3.3.1. 15-Lipoxygenase-inhibitor interactions

**Figure 2.**
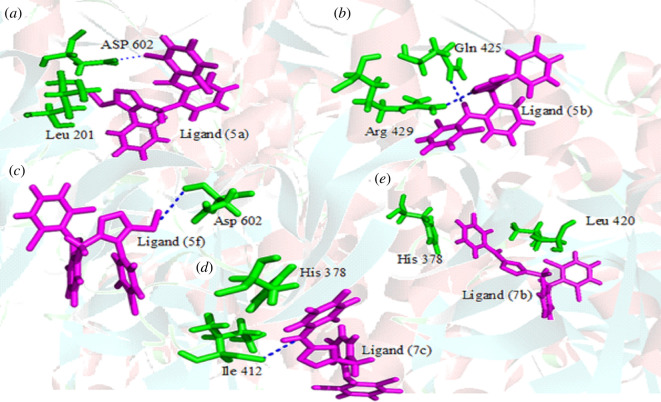
Protein–ligand interactions of compounds against the 15-LOXh. (*a*) The three-dimensional binding mode of most active compound **5a**, (*b*) for compound **5b**, (*c*) for **5f**, (*d*) for **7c** and (*e*) for **7b**.

To explore binding modes of diclofenac acid derivatives with human 15-LOX (15-LOXh), all the active compounds were docked against the 15-LOX. In the whole series, analogue **5a** to **5f** showed good docking scores and interactions with active site residues of 15-LOXh. The docking conformation of the most active compound in the series, compound **5a** showed good interactions with the active site residues of 15-LOXh. This compound established two cases of strong hydrogen bonding with active site residues, Asp602 and Leu201 of 15-LOXh. Asp602 showed interaction with chlorine atom whereas Leu201 exhibited interactions with the triazole ring of the compound. Further, this compound also showed hydrophobic interactions with the non-polar residues of the enzyme (figure 2*a*). The strong bonding network of this compound with active site residues of the enzyme might be one of the reasons for this compound depicting good inhibitory activity. Like compound **5a**, compound **5b**, the second most active compound in the series, also demonstrated a good docking score and good interactions with active site residues Gln425 and Arg429 of 15-LOXh (figure 2*b*). In the case of compound **5f**, it showed a hydrogen bond with active site residue Asp602 of the enzyme (figure 2*c*). The docking score observed for compound **5f** was less than **5a** and **5b,** probably due to the presence of chlorine instead of fluorine present on the phenyl ring of the compound. Further, the position of fluorine atom on the phenyl ring might also play a role in the activity and interaction of these compounds. For example, fluorine at ortho position (**5a**) enhanced inhibitory potential compared with when it is at meta position of phenyl ring of the compound (**5b**). The docking results of **7c** showed good interactions as compared with **7b** that might be due to the position of fluorine atom on the phenyl ring. The docking conformation of **7c** established one strong hydrogen bond with the active site residue Ile412 (figure 2*d*). Besides hydrogen bonding, this compound also formed pi–pi interaction with active site residue His372. In the case of **7b**, the docking results showed that only two pi–pi interactions were observed but no hydrogen bond was established by this compound with active site residues (figure 2*e*). Overall docking results show that the position of fluorine atom on phenyl ring depicted good interactions and activities as compared with the compounds having chlorine atoms on the phenyl ring [45].

#### 3.3.2. α-Glucosidase inhibitor interactions

**Figure 3.**
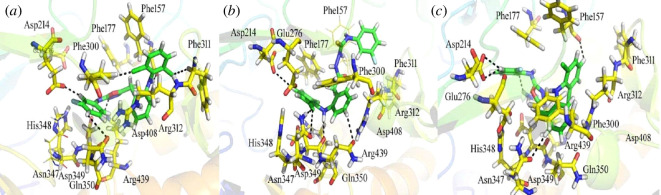
Docking conformation of compound **7b** (*a*), compound **4b** (*b*) and compound **7a** (*c*) in the active site of α-glucosidase.

The experimental findings were extremely well correlated with the docking results of the synthesized compounds with the α-glucosidase enzyme, which provided valuable information about the nature of the binding mode. The top-ranked conformations of almost all compounds were found to be well accommodated inside the active site of the α-glucosidase enzyme and to be involved in a variety of interactions with the target enzyme active site residues, according to the docking calculation study. It was discovered that compound from the compounds’ docking conformation **7b** (IC_50_ = 3± 1 µM, docking score = −13.6921) formed seven hydrogen bonds, one arene-cation and two pi–H linkages with the residues Phe 157, Glu 276, Phe 300, Phe 311, Arg 312, Asp 349, Asp 408 and Arg 439 of the α-glucosidase’s binding pocket. Phe 157 interacted as an H-donor with the 2,6-dichloro-N-(o-tolyl)-aniline anisole moiety of compound **7b**. It was observed that Glu 276 established H-donor interaction with the carbon atom of the fluorobenzene moiety of the compound **7b**. Asp 349 and Asp 408 made H-donor linkages with the –NH and carbon atom of the benzene ring of the 3-fluoro-N-methylaniline moiety of the ligand respectively. Arg 439 formed H-acceptor bond with the nitrogen atom of the 2-methyl-1,3,4-oxadiazole moiety of the compound as shown in figure 3*a*. Phe 300 and Phe 311 were observed making H-acceptor interactions with the chlorine atoms of the 1,3-dichlorobenzene moiety of the inhibitor, respectively, while Arg 312 residue formed pi–cation and pi–H contacts with the ligand. The inhibition of this compound might be due to the availability of the electron withdrawing groups (Cl, F and –NH) and electronic cloud system could be the cause of the compound’s excellent biological activity. Compound **4b** was the second most active (IC_50_ = 5 ± 1 µM, docking score = −12.1174) and showed five polar interactions and two pi–H, one H–pi and one arene–arene linkages with the active residues (Phe 157, Asp 214, Phe 300, Asn 347, His 348, Asp 349 and Gln 350) of the binding pocket as shown in the figure 3*b*. Electron donating groups (EDG) enhance the potency of the ligand (sulphur atom) as well as electron withdrawing groups (Cl, F and –NH) and delocalized electrons of the ligand. **7a** was found to be the third most active analogue (IC_50_ = 7 ± 1 µM, docking score = −12.1094). It was observed that this ligand formed five polar bonds, two pi–H and one pi–pi linkages with the active residues of the protein as shown in figure 3*c*. The potent inhibitory activity of this compound may be due to the presence of the electron withdrawing groups (Cl, F and –NH) and electron cloud system of the inhibitor, which create an electron flow making the compound more active, polarizable and potent. All the analogues of the synthesized series had good inhibitory activities as compared with the standard acarbose compound (IC_50_ = 376 ± 1 μM, docking score = −4.8720). Acarbose forms relatively strong hydrogen bonds when compared with other compounds, but because of its large size, it occasionally collides with catalytic residues. As a result, these conflicts make it less active than other compounds in the series (table 2). The docking score supported these findings as well.

## 4. Conclusion

In conclusion, our study has successfully synthesized and screened diclofenac acid derivatives, revealing their significant inhibitory effects on 15-LOX and α-glucosidase enzymes. These findings hold great promise for clinical application. Compounds **5a, 5b** and **7c** emerge as lead candidates for developing anti-inflammatory agents targeting 15-LOX, potentially offering effective treatment options for inflammatory disorders. Similarly, compounds **7b, 4b, 7a** and **8b** hold potential for managing diabetes by inhibiting α-glucosidase activity, which could lead to improved glycaemic control and reduced diabetes-related complications. These derivatives also demonstrate minimal toxicity to blood mononuclear cells, indicating a favourable safety profile. Furthermore, molecular docking studies provided mechanistic insights into key interactions with the enzymes’ active sites.

To further validate the therapeutic potential of these derivatives, additional *in vivo* studies are warranted. Clinical trials will be crucial for assessing their efficacy, safety and suitability for patients with inflammatory disorders and diabetes. Ultimately, our research paves the way for the development of novel therapeutic agents with real-world applications, offering hope for enhanced treatment strategies and improved patient outcomes.

## Data Availability

The datasets supporting this article have been uploaded as part of the electronic supplementary material [49].
